# Strategy inference during learning via cognitive activity-based credit assignment models

**DOI:** 10.1038/s41598-023-33604-2

**Published:** 2023-06-09

**Authors:** Ashwin James, Patricia Reynaud-Bouret, Giulia Mezzadri, Francesca Sargolini, Ingrid Bethus, Alexandre Muzy

**Affiliations:** 1 Université Côte d’Azur, CNRS, I3S, Nice, France; 2 Université Côte d’Azur, CNRS, LJAD, Nice, France; 3 Columbia University, Cognition and Decision Lab, New York, USA; 4 Aix Marseille Université, CNRS, Laboratoire de Neurosciences Cognitives, Marseille, France; 5 Université Côte d’Azur, CNRS, IPMC, Nice, France

**Keywords:** Neuroscience, Mathematics and computing

## Abstract

We develop a method for selecting meaningful learning strategies based solely on the behavioral data of a single individual in a learning experiment. We use simple Activity-Credit Assignment algorithms to model the different strategies and couple them with a novel hold-out statistical selection method. Application on rat behavioral data in a continuous T-maze task reveals a particular learning strategy that consists in chunking the paths used by the animal. Neuronal data collected in the dorsomedial striatum confirm this strategy.

## Introduction

To learn complex tasks, individuals (human or animal) often decompose them into elementary sub-tasks and learn an execution order for these elementary sub-tasks (also called actions in reference to reinforcement learning)^1^. This decomposition can be more or less coarse. For instance, when children learn how to go to school by themselves, do they segment their path at every turn, or do they employ a coarser representation such as “go to the bakery” (because they have done it so many times with their parents) and then “turn left, the school is at the end of the street”? More generally, what strategy is at play when learning complex tasks? By strategy, we mean here a representation of the world in terms of states and possible actions to be performed in each state. Inferring this representation would reveal how individuals understand their environment and their actions, and could help to shed light on covert (neuro)cognitive processes. This strategy can be based on an action representation, *i.e.*, a chunking/grouping of actions as above^1–3^, or a state representation, where only certain features are important, *i.e.*, rule-based reasoning (“stop when the pedestrian light is red”)^4^; it can also involve actions based on the similarity of the states, *e.g.*, reasoning based on similarity (“would rather walk on large sidewalks like the one at home than on small ones”)^5^, etc.

The purpose of the present work is to infer a learning strategy. In the sequel, learning always refers to a cognitive process, that is the learning of a given task by an individual (or the corresponding model), whereas inference always refers a statistical procedure (eventually combined with an experimental protocol) to guess the learning strategy. The two contributions of this work consist of: (i) a new inference procedure to test different models or hypotheses about how agents learn using only individual observed choice behavior, and (ii) a new version of Activity-Credit Assignment^6^, named Cognitive ACA (CoACA), to model the different possible cognitive strategies used during the learning. In short, we describe strategy-based learning models and an inference procedure - based on cross validation - to select the best model based upon empirical observations.

Many cognitive experiments in human or animal setting have been designed to infer particular features of strategies, *e.g.*, the allocentric/egocentric spatial representation^7^. Most of the time, the design of these experiments is such that the representation is tested at the end of the learning, making it difficult to infer the strategy during learning. These tests are typically called probe tests for animals or transfer phase for humans^8–10^ and consist in showing new stimuli/situations without giving feedback or reward. Some experimental designs use probe tests during the learning phase^11^. While this can reveal a clearer picture of the strategy during learning, it has been shown that these tests, which interrupt learning, can also alter the learning itself^12–14^.

Therefore, if one wants to understand how a human or animal chooses one strategy or another during free learning (*i.e.*, learning that is not modified by any external intervention except rewards/feedbacks), one must look for experiments that are not hindered by probe tests. This is especially true for continuous spatial tasks. For example, by allowing mice to freely learn sequences of positions in an open space, Belkaid et al.^15^ showed that the best description of learning, in mice, was afforded by changes in the degree of random exploration by estimating the degree of randomness at different stages of learning. However, most of the time, strategy inference cannot be reduced to the estimation of one parameter in a sole model. Indeed, typically in a maze, the different strategies are granular (discretization at each junction). In this setting, auxiliary data are generally used to deduce some characteristics of the strategy. For example, in van der Meer et al.^16^, the authors use very fine-grained behavior, namely forward-backward movements at Junctions, called Vicarious Trial Errors (VTE), to determine where the decision is made^17^. Neural data can also be used: neural trajectory decoding techniques have suggested that trajectories are chunked using particular brain states (theta cycle length and number of gamma cycles per theta cycle)^18^.

The goal of the present work is to propose a much more general method for inferring strategies during learning that could deal with model selection. Our method is based only on free learning data, without the need for probe tests or auxiliary variables (such as VTE or neural data). It can be applied on an individual-by-individual basis and thus reveal inter-individual variability. Indeed, the strategy may vary from individual to individual and averaging across individuals could lead to misleading conclusions^19–22^. The method proposed here is validated only in a second step, by neural evidence recorded on animals (in the present application) at a later stage, but it could also be validated by other auxiliary data. Note, however, that this validation is not part of the method itself in order to keep the method as general, flexible and agnostic as possible.

To this end, the method proposed here is based on a specific hold-out statistical method that is adapted to the learning data of a single individual and requires no prior knowledge.

Estimation in learning has a long history and model selection is usually done either by cross-validation on a population of individuals, which assumes the existence of a shared strategy, or by Bayesian model selection, which requires prior constraints on the parameters of a model or, indeed, the model itself. However, these priors are not universally available. For more details, we refer the readers to Daw’s review^23^ and the references therein, but also to Collins and Wilson’s crystal clear methodological work giving “10 simple rules” to follow for performing computational modeling of behavioral data^24^. Here, we focus on a hold-out method that we have specifically designed to select learning models based on a single learning experiment of a single task.

Cross-validation (and hold-out as one of its special instance) is a very old statistical method that seems to date back to the 1930s^25^. It consists in separating data into two sets: a training set to train the models (*i.e.*, estimate the parameters) and a testing set to evaluate the models fit, these two sets being generally independent. When the fit is evaluated by log-likelihood, the difference of log-likelihood can be seen as the logarithm of a likelihood test ratio between two models or as a crude (log)Bayes factor^26^. The current hold-out method is a continuation of a previous work on human category learning^11^, where two models involving different learning strategies were pitted against each other: Alcove^5^, in which individuals learn by similarity, and Component-Cue^4^, in which individuals learn by features or rules. To do this, we interleaved the learning phases with several (human) probe tests, using the probe tests data as the testing set for the hold-out method. However, the use of this specific interleaved protocol might have changed the free learning strategy as mentioned above^13^. Here, we take this a step further by showing that hold-out can work even on the (dependent) learning data of a single individual, without having to perform specific experiments.

This statistical method is then demonstrated with a particular type of Reinforcement Learning (RL) algorithm called *Activity-based Credit Assignment* (ACA)^6^, from which several new versions are derived to model different cognitive strategies. Several RL algorithms, called hierarchical abstract machine^27,28^ or hierarchical reinforcement learning^3^, have been designed to decompose tasks into sub-tasks (see also Botvinick et al.^2^). We decide to consider an ACA algorithm^29^ that is much simpler and more modular than previous hierarchical RL algorithms in order to ensure a robust statistical fit of the data without risking overfitting. From a modeling point of view, it also has the advantage of easily incorporating certain characteristics of the actions, such as their duration, into the notion of activity.

Our inference strategy can be used in a second step to better understand other data, typically neural data and as an application, we show that the strategy selected by our method matches the neural data in a continuous T-maze alternation task.

## Results

### Description of the method

The method consists of the following steps:

1. Choose the different strategies that one would like to put in competition and model them. We here develop a cognitive variant of ACA, featuring only behavioral variables and named CoACA. The latter is based on two main notions:
  A credit *K*(*S*, *A*) which is the credit of action *A* when the individual is in state *S*. At each new state *S*, the action *A* is selected at random (the action with the highest credit being the one that is selected more often). Defining the set of possible states and actions determines the level of chunking, or more generally the strategy. For instance, in the toy example of children learning to go to school, the actions can be all the possible turns (left, straight and right) leading to the school and the states can be all the possible crossroads. Another potential chunking can be defined by a longer and more complex action such as “go to the bakery”, whereas the states can be reduced to “at the bakery” and “at school”.
  An episode, which is a group of actions that have to be executed before evaluating the reward. These actions may have to be performed sequentially (for instance for the children’s path), or the order may not matter (for instance if we add “take the keys”, “take the backpack”, etc.). Whatever the chunking, the notion of episode usually does not vary, since it ends when individuals have all the information to adequately estimate their reward. For instance, for the children an episode would end when coming back from school, since their highest reward would be to have been to school and to have their keys to go back home.
  For a given episode, a reward *R* is obtained. Then, we update the credit for all the actions *A* that have been performed during the episode:
  1 $$\begin{aligned} K(S,A)\leftarrow K(S,A)+\alpha ~a(A) R, \end{aligned}$$
  where $\alpha \in [0,1]$ is a learning parameter and *a*(*A*) is a measure of the activity corresponding to action *A*. This activity might reflect several features, for instance the fact that a longer action might be more important and therefore would have a larger activity in order to give more credit to this action.
  Other features might be added: for the children’s example, one could add forgetting after the vacations, which would lead to a decay of all credits right after the vacations.
  Also, CoACA is more than a chunking tool. For instance, and inspired by Component-Cue^4^, we could design a credit of the form
  $$\begin{aligned} K(S,A)=K_1(S_1,A)+K_2(S_2,A), \end{aligned}$$
  where $S_1$ and $S_2$ are two features of the state *S* and the update (1) is not performed on *K*(*S*, *A*) but on each of the credits per feature $K_1(S_1,A)$ and $K_2(S_2,A)$. Both of them could be updated by different states sharing the same feature (for instance the presence of the red pedestrian light) and would explain a learning where states with the same features have the same pattern of likely actions. However, in the present work we only focus on chunking models, that are defining different sets of states and actions, using (1) directly.
  **NB:** In the sequel, we will use indifferently “models” and “algorithms” to refer to this step, that is the specification of all the states, actions, and parameters involved in the update of the credit. Note that if their number is fixed at this stage, the value of the parameters for a given model are not known. This will allow us to fit the model on the data.
2. Once all the strategies in competition (represented by different models, typically modeled by different CoACA algorithms) have been designed, estimate the parameters (at least the learning parameter $\alpha$) in the *early learning phase*, which represents the training data set. The early learning phase consists of all the actions taken between the beginning of the experiment and a given point in time to choose. The estimation in the early learning phase is performed by maximum likelihood (see Daw^23^ for an excellent review of likelihood estimators in RL algorithms). The models with estimated parameters are called the trained models, each of them corresponding to one strategy.
3. Select the strategy which corresponds to the trained model with the maximum likelihood in the *late learning phase*, that is the complement of the early learning phase in the data. This late learning phase corresponds to the testing data set of the hold-out procedure.

As compared to Mezzadri et al.^11^, we do not use data without feedback as the testing set. Instead, we use the late learning phase as testing set to provide an approach that can be applied to a larger panel of experiments. Note that it would not have been possible to easily train the models on the late learning phase or perform other forms of selection (such as the cross-validation^30^), since individual learning data are not stationary. Indeed, learning algorithms always update the probability of the choice of a given action as a consequence of the feedback or rewards and as a function of the non-observed parameters, but it usually starts with equal initial probabilities to select a given possible action in a given state. Hence, it is easy to start with one set of parameters, compute the evolution of the probability on the real data set, and find the parameter that is the closest to the data (by maximum likelihood estimation). On the contrary, if the estimation is not performed on the early learning phase, one would have to guess or estimate with which probability the learning algorithm starts its evolution, which would have been much more intricate and computationally costly.

Another difficulty for inferring strategies in learning experiments is that the late learning phase might not contain enough information, depending on the models. Typically, if all the strategies in competition lead to learning algorithms that are so good that they do not make any mistake in the late learning phase, and if the corresponding data are also without any mistakes, it is not possible to select anything meaningful (see Material and Methods and SI).

Therefore, we need a thorough simulation study to be sure that the models and the partition between early learning phase and late learning phase are done in such a way that the whole procedure does indeed identify a winning model efficiently. The early learning phase needs to be long enough to guarantee a good estimation of the parameters, but the late learning phase needs to still contain enough information to discriminate between models. A trade-off needs therefore to be found in simulations (see Material and Methods and SI). Note that the forgetting aspect might also play a crucial role. Indeed, from a data standpoint, it might be helpful to incorporate it to obtain a more realistic algorithm. In addition, forgetting might lead to more errors in the models in competition and therefore help the hold-out procedure because there is still enough information in the late learning phase.

### A continuous T-maze spatial alternation task

To illustrate the previous method, let us now describe a particular learning experiment.

**Figure 1:**
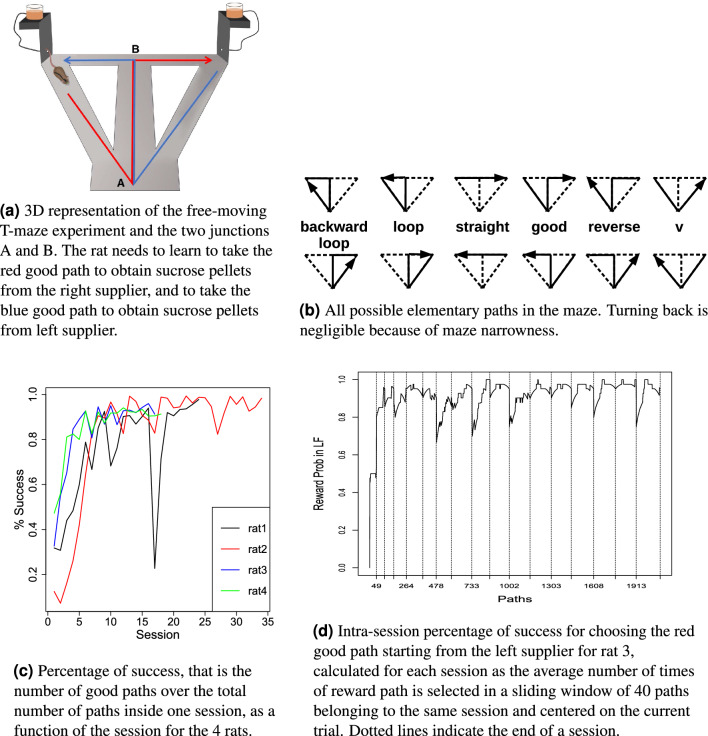
Experimental protocol and learning curves.

The experiment consists in a modified ‘T’ maze apparatus^31,32^, where rats learn to take specific good paths indicated in Fig. 1a to find sucrose pellets at one of the two suppliers. Four adult rats (Long Evans, Janvier) were used in this experiment. Single Unit activity of neurons in the dorso-medial striatum was recorded during a 20mn freely moving session per day. Each rat performed at least 18 sessions and at most 51 depending on the quality of neural recordings.

We identified 12 possible elementary paths that rats traveled during the different sessions. Since the maze arms were narrow (12cm), ‘turn back’ paths were extremely rare and therefore were not included among the elementary paths (see Fig. 1b). In the sequel, the word “path” only refers to one of the twelve elementary paths (see Fig. 1b), neglecting all other types of behaviors. A session is therefore a long sequence of paths from one supplier to the same supplier or to the opposite supplier. Figure 1.c shows the percentage of success, that is the number of good paths (left or right, see Fig. 1b) in one session divided by the total number of paths taken inside this same session, as a function of the session and the rat. Figure 1d shows more precisely the intra-session evolution of the percentage of success of rat 3 for the paths starting from the right supplier: declines in performance are appearing mostly at the beginning of each session. The curves for every rat (not reported here) were very similar. One of the many explanations for these drops could be forgetfulness.

### CoACA models of the free-moving T-maze experiment

Our main goal on the T-maze experiment is to understand whether the rat plans its trajectory as a whole and somehow knows from the start (when leaving a supplier) how it will go to the other supplier, or if it decomposes this path into smaller elementary chunks.

Before detailing the different chunking models, let us first detail the common aspect of each of these models.

**Figure 2:**
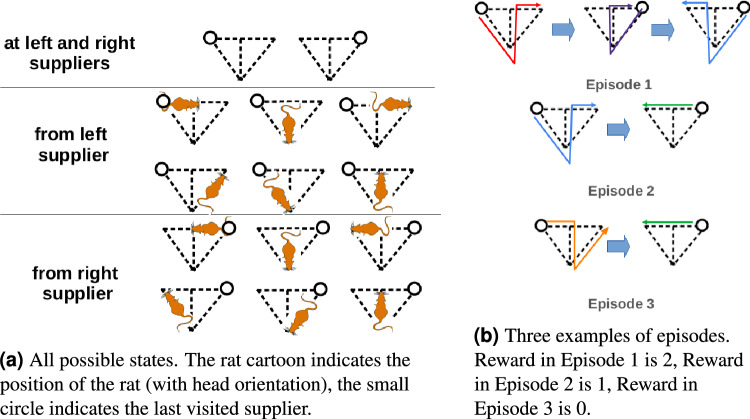
All possible states and examples of episodes.

A state (see Fig. 2a) is defined by the current position of the rat in the maze and the last supplier it visited. Indeed if a rat does not remember the last visited supplier, it cannot distinguish the red good path from the loop once it is in the central stem. This is not important of course if the path is planned as a whole but, if it is chunked in smaller pieces, this information has to be kept in the model.

An action is a path (see Fig. 2a) or a chunk of a path (cutting could be done at junctions A and/or B, see Fig. 1a). An episode consists of starting from one supplier, going to the other supplier at least once, and then returning to the original supplier. Indeed rats were habituated to the environment and knew that food could come from both suppliers. It is therefore reasonable to think that the correct evaluation of the reward would take place only once both suppliers have been visited at least once. In particular an episode could be constituted of a good path, a loop and the other good path (the reward is 2) but also, for instance, by a good path, and a wrong path to come back (the reward is 1) or two wrong paths (the reward is 0) as shown in Fig. 2b. The duration of an episode in terms of number of paths or even actual duration is therefore not fixed.

The activity of an action is the percentage of time during the whole episode that an action takes. The idea is that the shorter the action, the less the rat is involved in the action.

Finally all credits *K*(*S*, *A*), for all actions *A* and states *S* are multiplied by a factor $\gamma \in [0,1]$ at the beginning of a session (or equivalently at the end of the previous session) to model the forgetfulness of the rat from one day to another.

Therefore each model is parameterized by $\theta =(\alpha ,\gamma )$, the learning parameter and the forgetfulness parameter.

**Figure 3:**
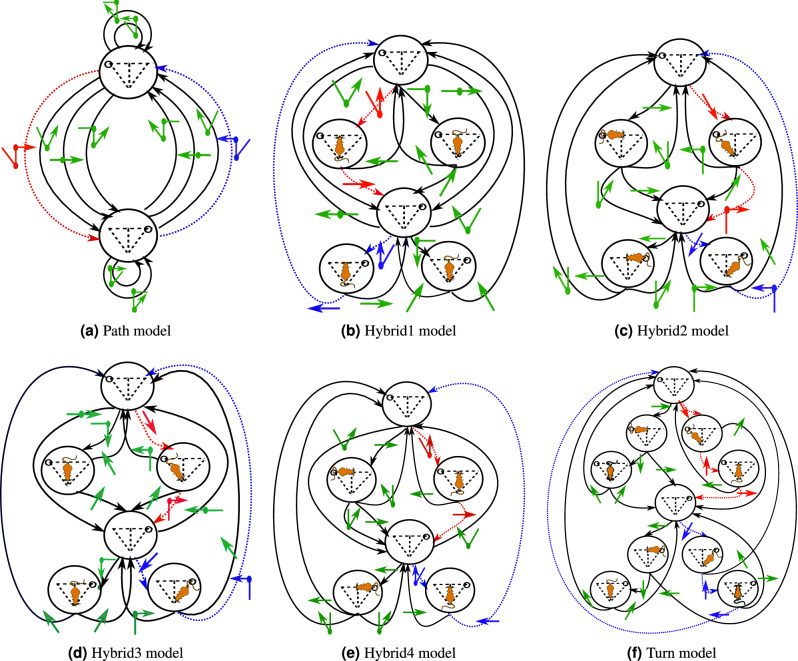
Decision diagrams of the 6 different CoACA models. Nodes of the graphs are the possible states, edges corresponds to the possible transitions from one state to another, the actions are represented by colored arrows on top of the edges. In red, the actions corresponding to the red good path of Fig. 1a. In blue, the actions corresponding to the blue good path of Fig. 1a. In green, actions constituting the other paths.

Let us now explain what are the different models (see their corresponding diagram in Fig. 3). In the model with the smallest number of states, we consider as an action, a path in the sense of Fig. 1b. This is the Path model that has only two states (the two suppliers) and from each state, 6 possible actions (the six paths starting from one supplier) (see Fig. 3a).

The model with the largest number of states consists in considering that an action is just a turn and that the action is selected at random (according to the credit) at each junction. This is the turn model, with 10 states and two possible actions in each state (see Fig. 3f).

In between these two extremes, the rat can have different strategies:

- The rat deliberates only at the second encountered junction if there are two of them in its path, *i.e.*, the model picks the last part of the path according to ACA rule (Hybrid 1 model, Fig. 3b).
- The rat deliberates at the first encountered junction (Hybrid 2 model, Fig. 3c). This reflects more or less the fact that the rat does not need to plan everything in advance. It moves forward and then decides at the first junction it encounters what it has to do next.
- It is also possible that this is not the first encountered junction that matters but a physical Junction A or B (See Fig. 1a), where the rat is making its decision (that is the model picks the action at random according to ACA rule). If this is A, this leads to the Hybrid 3 model (Fig. 3d). If this is B, we obtain the Hybrid 4 model (Fig. 3e).

### Strategy inference on the T-maze experiment

After a complete simulation study (see Material and Methods and SI), we have shown that defining the early learning phase as the first 800 paths that have been taken and the late learning phase as the paths from 801 to the end of the experiment for each individual guaranteed a good accuracy of the method. In particular, this simulation study shows that, if the rat was indeed behaving like one of the 6 models, the method is able to identify the correct model in more than 67$\%$ of the simulations (this happens in only 1 over 22 cases, the other 21 cases having a percentage of correct identification larger than 83$\%$).

Next on the real data set, Table 1 gives the log-likelihood of the trained models on the testing data set, for each rat.

**Table 1:** Comparison of the log-likelihood of the trained CoACA models presented in Fig. 3 for the 4 rats.

Rat	Models						
	Paths	Hybrid1	Hybrid2	Hybrid3	Hybrid4	Turns	Selected
Rat 1	− 841.82	− 1198.88	896.16	**−739.04**	− 1370.52	− 1170.65	Hybrid3
Rat 2	− 916.02	− 1484.91	− 932.43	**− 878.77**	− 1479.51	− 3947.30	Hybrid3
Rat 3	− 456.90	− 725.83	− 459.94	**−446.99**	− 774.72	− 623.06	Hybrid3
Rat 4	− 722.94	− 1067.23	− 726.10	**−715.69**	− 1116.30	− 969.62	Hybrid3

In bold the maximal values. Note that the smallest difference in log-likelihood is 7.25 and is achieved for rat 4 between Paths and Hybrid 3. This difference means that the likelihood test ratio between Hybrid 3 and Paths is about 1400.

Therefore all the rats seem to learn according to the Hybrid 3 model, which means that their learning is driven by a chunking of their path. Since the Path model is not chosen, it means that they do not seem to plan the whole path when leaving a supplier but to fragment it. The fact that the Turn model is not chosen as well means that they do not need to stop at each crossroad either. All the rats seem to deliberate at Junction A in the maze (Hybrid 3 model) (see Fig. 1a). Note that from the criterion point of view, Paths, Hybrid 2 and Hybrid 3 have very close values, even if Hybrid 3 is the chosen model, whereas models Hybrid 1, Hybrid 4 and Turns are much less likely.

### Neuronal validation of the selected chunking in the T-maze experiment

Our strategy inference method is self-consistent and does not need auxiliary data *per se*. But, it can provide a new way to look at neuronal data and new neuronal evidence that the inferred strategy (namely Hybrid 3) has a neuronal counterpart.

Using DorsoMedial Striatum (DMS) lesions, we have shown in a previous work that the DMS is essential in the learning process of this spontaneous spatial alternation task^31^. Hence, if we consider that the neuronal activity in DMS encodes the behavior, neuronal activity should be different when the rat knows where it wants to go. More precisely, if at a given location, for instance the central stem of the T-maze, the future path is known by the rat, neurons should be coding a difference between turning right and turning left. Coding is here understood as a significative statistical difference between the firing rates in both conditions (see Material and Methods).

**Figure 4:**
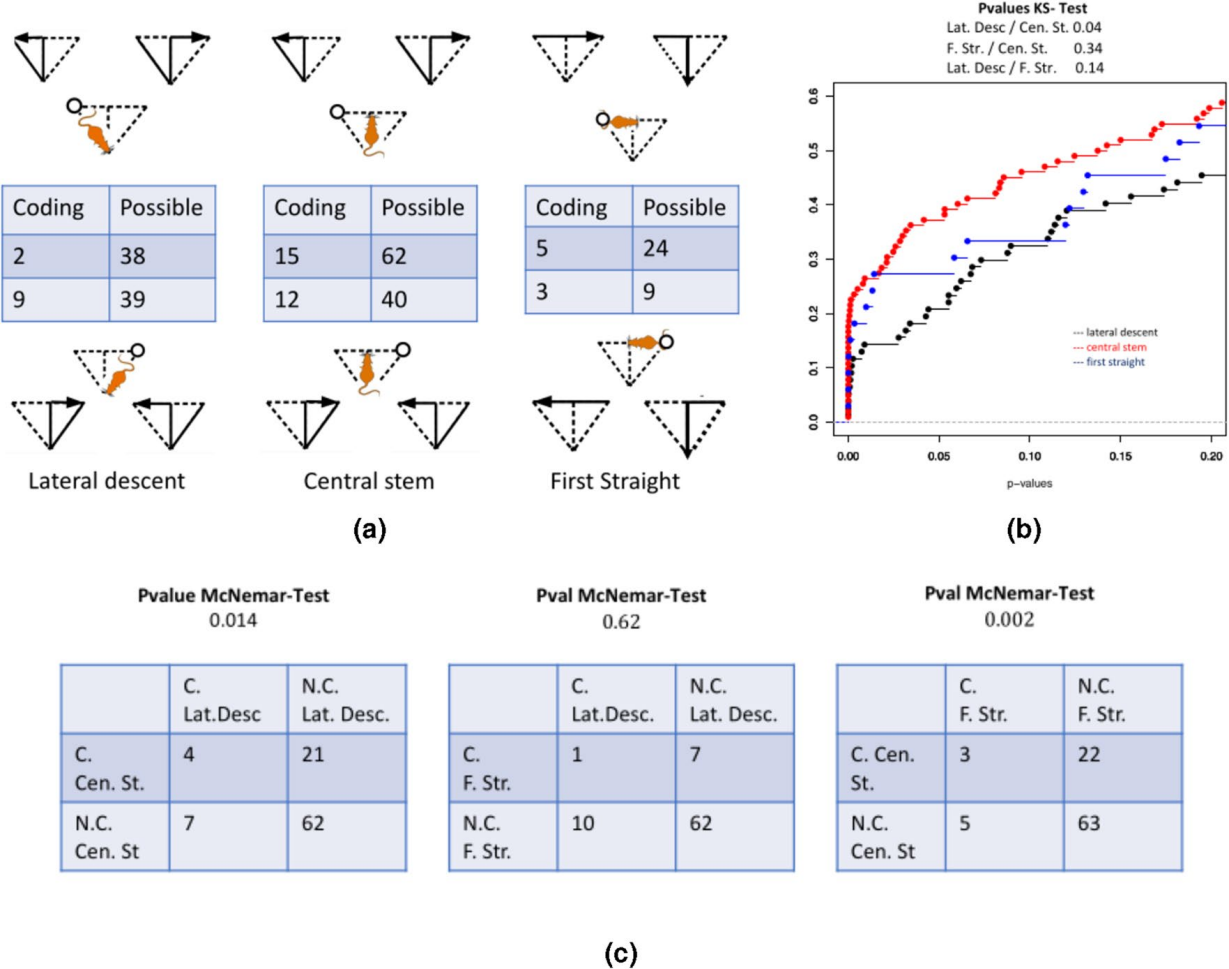
Analysis in terms of firing rate coding neurons. In (**a**) all neurons of all sessions are analyzed in terms of their firing rate. If the firing rate in a given location between two possible future (sub)paths is sufficiently different (see section “Methods”), the neuron is said to be coding. This analysis is possible only if the two conditions are present in the session, when the neuron has been recorded and if the neuron fires enough inside the location under consideration. The number of neurons for which the computations were possible is given as well as the number of coding neurons among them. $3\times 2$ different locations (named respectively *Lateral descent*, *Central Stem* and *First Straight*) have been tested, they are indicated for the first row (resp. second row) by the position of the cartoon rat is the maze on top (resp. bottom). The two different future (sub) paths that are compared are given on top (resp. bottom). The difference between the first and second rows is the location of the last visited supplier (right and left). In (**b**) the firing rates being highly inhomogeneous among neurons, we computed for each of them a p-value of the test of equality of the firing rates between the two future (sub) paths. The cumulative distribution functions (zoomed on small values) of the pvalues of each of the three conditions are plotted (red for central stem, blue for first straight and black for lateral descent, right and left are pooled). All c.d.f. are non uniform (Ks Test of homogeneity with pvalues smaller than $2.10^{-5}$). Coding neurons counted in (**a**). are the ones for which the adjusted Benjamini-Hochberg pvalues is smaller than 0.05 (the multiplicity correction is applied for all the possible neurons at a particular location). Also corresponding p-values of Kolmogorov Smirnov tests of equality of the c.d.f. are given on top. In (**c**), comparison of the number of coding neurons (**c**) and non coding neurons (N.C.) between two particular locations (right and left are pooled). McNemar tests have been performed to decide if the diagonal elements of the tables are statistically different and p-values are written on top.

First, Fig. 4a shows that we found coding neurons at each of the 3 locations that have been tested: lateral descent, central stem and first straight. This can also be seen on the 3 cumulative distribution functions (c.d.f.) of the p-values for the test of equality of the firing rates between the two conditions (Fig. 4b) which are all significantly non uniform (p-value $<2.10^{-5}$).

However, in all the data that have been collected only eleven neurons are coding at the lateral descent location, whereas almost three times more are coding in the central stem. This difference is significant. Indeed, the c.d.f. of the corresponding p-values for the central stem is clearly above the one for the lateral descent (p-value of the KS test 0.04) and the McNemar test of equality of the two proportions has a p-value of 0.014 (Fig. 4c). This leads us to think that the cognitive state in the lateral descent is different from the cognitive state in the central stem and that there is much more coding in the central stem.

It also tells us that in the central stem, there is a significant number of coding cells that have a different firing rate between turning left or right (that is a loop or a good path). This should mean that the rat would know where it will turn before Junction B (see Fig. 1a).

Both of these facts are only coherent with strategies Hybrid 2 or 3, among all the 6 strategies.

To distinguish between Hybrid 2 or 3, we can look at the difference of cognitive states in the first half of the straight path before Junction A. We find here again some coding neurons. The c.d.f. of the first straight (in blue) seems to be clearly higher than the lateral descent, and would lead us to think that there is indeed a strong anticipation of the next move before arriving to Junction B, fact which is only coherent with Hybrid 3. However, we did not find any statistical evidence (p-value of 0.14 for the KS test and 0.62 for for Mc Nemar test). This might be due to the relative small number of neurons for which the test can be performed between straight paths and turns at Junction B. Indeed, we need sessions where the rat turns sufficiently many times in the middle to compute these statistical tests and if the straight path is frequent, it is not very frequent to have turns towards the central stem at Junction B (see Material and Methods for more details).

Note however that we have evidence that the coding is stronger in the central stem than in the first straight (p-value 0.002 for the McNemar test). Also note that we also find some coding neurons in the lateral descent, which would lead to think that the rat might be aware of the whole path since the lateral descent, even if it is less strong that in the central stem.

To summarize, we have strong neuronal evidence against Turns, Hybrid 1 and 4, because none of them would explain such a strong anticipation in the central stem. This is in adequation with the fact that the negative log likelihood of these 3 models are very large (see Table 1). The fact that there is much less coding in the lateral descent than in the central stem, makes us reduce the possibilities to Hybrid 2 or 3, even if it is possible that the rat might plan its whole path, since very few neurons are coding in the lateral descent. Finally, because there are also neurons coding in the first straight half, it would lead us to privilege Hybrid 3, but the statistical evidence is tempered by the small amount of neurons supporting this fact. This also explains why we can do this neuronal analysis only on pooled data of all the 4 rats and when looking at pairs of paths where at least one of them is good or straight (that is the most likely behaviors).

## Discussion

In the present work, we infer strategies, that is representations of the states and actions, used during a learning task and this individual per individual. This relies on two main ingredients: a specific hold-out method and particular learning models.

First, we develop a hold-out procedure that works even if the learning data are neither independent nor identically distributed. This seems to be in contradiction with the excellent review by Daw^23^, which advises against this method in non-i.i.d. situations and prefers Bayesian procedures (see also Wilson and Collins^24^ for the use of the Bayesian Information Criterion (BIC)). However, Bayesian procedures require an *a priori* knowledge that we do not have here, and the BIC criterion would be useless when the competing models have the same number of parameters. But, under certain specific conditions, hold-out can give good results even in non-i.i.d. contexts. Indeed, a very recent work^33^ has proved mathematically, via oracle inequalities, that hold-out can work on Markov processes. Even if we are not strictly speaking in their mathematical framework, the CoACA models are essentially Markovian if we examine them episode by episode. Simulations on a realistic set of parameters, following the rules of Wilson and Collins^24^ (see “Material and Methods” and “SI”) have shown that our specific hold-out procedure is able to identify the CoACA model in more than 67% of the simulations (this happens in only 1 out of 22 cases, the other 21 cases having a percentage of correct identification higher than 83%). Therefore, our first contribution is to say that hold-out can work on individual learning data if the models are “sufficiently identifiable”.

By sufficiently identifiable, we mean that there is still enough information in the late learning phase, which we use as testing set, to infer the model and thus the strategy. The CoACA models we used, especially with their session-to-session forgetting aspects, have this property as shown in the simulations. Moreover, they are able to model simply the different strategies we want to compare.

While the fitting of RL algorithms to learning data has a long history^15,23,24^, it has been less used to infer strategies, in the sense of granular representation of state and actions. Indeed, there are many RL algorithms, different from CoACA, that are capable of modeling different strategies: hierarchical abstract machines^27,28^, hierarchical reinforcement learning^3^, etc. However, most of the time, these models are not used to fit data but to prove the optimality of a given strategy in a certain sense. For example, in Solway et al.^3^, a given hierarchy model, which is equivalent to a chunking in our framework, defines small reinforcement learning problems on each subtask. The authors computed the optimal hierarchy, as the hierarchy for which the overall algorithm is most likely to obtain the best reward. Because the authors were interested in human tasks, they were able to design experiments in which it was easy to determine which hierarchy was used by individuals by asking them auxiliary questions. As a result of their work, it appears that humans use the optimal hierarchy in various tasks such as delivery problems, Hanoi tower, etc. But note that they inferred the strategy, not from the free learning data they had, but from an auxiliary survey that the participants answered.

The CoACA algorithms presented here, as cognitive versions of the ACA algorithms^29^, are simpler than the previous hierarchical RL algorithms and can incorporate additional information, such as forgetting, that could be useful from a modeling/fitting point of view. ACA also uses a particular notion of episode, different from the one classically used in RL^34^. In ACA, episodes consist of elementary groups of decisions after which a reward is integrated in the individual’s prediction. With respect to the strategy inference problem, each episode can be chunked in different ways, which lead to different versions of ACA. Furthermore, unlike hierarchical RL, ACA does not assume anticipation of rewards and does not consider subsequent rewards to be of less and less importance. In ACA, all actions in an episode are constituents that can all contribute differently depending on their own activity. The advantage of ACA is that, on the other hand, important characteristics of the actions (such as duration in the T-maze task) can be incorporated into the notion of activity: this allows the model to be more flexible, without greatly increasing the number of parameters.

Another trend in the literature, different from the strategy inference based on learning data, is the development of increasingly complex models of multiple cognitive structures and functions at the same time^35–37^. For example, typically in Dollé et al.^37^, this trend aims at gathering all the qualitative knowledge of the brain in a huge numerical model with a very large number of parameters that would be able to do “as well” as an individual performing free learning. However, it is difficult with so many components to discriminate which features are necessary to represent which behavior, and the model is so complex that it is almost impossible to fit the parameters to real data. In particular, one cannot make strategy inference with such a complex model.

That said, given all the existing literature on more complex models, it would certainly be interesting to incorporate more features into the competing models, for example by starting with a hierarchical abstract machine^27^, simply adding decayed reward anticipation^34^ or adding more variability in the decision^15^ and gradually increasing the complexity of the models. The hold-out procedure would then be able to detect the minimum complexity model that best reflects the available data. This must be tempered by the fact that the higher the number of parameters, the longer the early learning phase (training set) must be, in order to obtain a correct estimation of the parameters and to trust the inference method. Thus, although we do not believe that it is possible to achieve such a high complexity as Dollé et al.^37^, the selection of key features to incorporate is a promising avenue for future work. It would also be interesting to see how a strategy changes throughout the learning process as Belkaid et al.^15^ have done for the choice variability. However, the hold-out presented here would then have to be performed on several segments much smaller than the whole data set and it is not sure that the accuracy of the method would then be sufficient.

From the user’s perspective, the performance of our method (CoACA combined with hold-out) makes it a promising pre-processing of behavioral data. Thanks to the strategy it selects, it reduces the search for significant neural behaviors (see also Rule 9 *Analyze winning patterns* of Wilson and Collins^24^, where latent variables—here strategies—are linked to physiological data). The user is thus no longer doomed by an exhaustive search of significant neuronal differences in the space of all possible chunkings of space and time, whereas such exhaustive search would have to be corrected for multiple testing errors and would have therefore a very low detection rate. This approach is not limited to the continuous T-maze alternation task but could be extrapolated to other learning experiments.

More specifically, for the experiment presented here, we decided to record neural activity in the dorsomedial striatum (DMS) during the continuous T-maze alternation task since a lesion in this region impairs learning for this task. In the present work, we go further by showing that DMS neurons have a firing rate that is coding for action anticipation. However, other anatomical structures, such as the dorsolateral prefrontal cortex (see Botvinick et al.^2^ and references therein) or the dorsolateral striatum^38^ are also involved in the representation of the world in terms of states and actions. It is therefore legitimate to ask, for future work, how these two structures interact or share these representations, and this should be facilitated by the chunking of the behavioral data provided by the method presented here.

In conclusion, CoACA models are simple enough to be selected on real learning data of a single individual via a new hold-out procedure. This allows us to infer strategies, *i.e.* representations of the world in terms of actions and states, during the learning phase, without any *a priori*. This strategy inference then allows us to target a temporal or spatial window, during which the neural encoding/decoding aspects can be studied, and this method could be applied on a vast variety of learning experiments. Increasing gradually the complexity of the models in competition can also be envisioned.

## Methods

### Experimental design

#### Animals and surgery

Four male Long-Evans rats (Janvier, Le Genest-St-Isles, France) weighing 300–350 g were housed in individual cages (40 cm long $\times$ 26 cm wide $\times$ 16 cm high) with food and water ad libitum and maintained in a temperature-controlled room (20$^{\circ }$ + 2). One week after their arrival, animals were handled daily by the experimenter for 7 days. They were then implanted with tetrodes aimed at the dorsomedial striatum (DMS) at the following coordinates: AP: +1mm, ML: ±2.2 mm from the midline, DV: $-3$ mm below the dura. The surgery was performed under sterile conditions and under general anaesthesia (Ketamine 75 mg/kg (Imalgene 1000, Merial, France)/Medetomidine 0.25 mg/kg (Domitor, Janssen, France)). As postoperative treatment, the rats were injected with antibiotic (Clamoxyl, 150 mg/kg) and analgesic (Tolfedine, 4 mg/kg). After surgery, the rats were given 5–7 days of recovery. They were then subjected to a food deprivation program that kept them at 90 pourcent of their body weight to start the T-maze continuous task. All experiments were performed in accordance with the National Institute of Health’s Guide for Care and Use of Laboratory Animals (NIH Publication No. 80-23) revised in 1996 for the UK Animals (Scientific Procedures) Act of 1986 and associated guidelines or the Policy on Ethics approved by the Society for Neuroscience in November 1989 and amended in November 1993 and under veterinary and National Ethical Committee supervision (French Agriculture Ministry Authorization). Experiments were performed in the Laboratory of Cognitive Neuroscience (UMR7291) and protocols were approved by the host institutions: CNRS and Aix-Marseille University. We certify that all our methods are in accordance with the ARRIVE Essential 10 of the National Center for the Replacement Refinement & Reduction of Animals Research and our manuscript has followed the Arrive recommend set. Following the 3R we used only four male Long Evans animals. Since our study is about learning under physiological conditions, each animal is its own control and the behavior task has been done on the same animal implanted with the headstage for the single unit recording system. Each animal is in an individual cage because of its headstage and to avoid any damage to the animal. Surgery has been done in accordance with the National Institute of Health’s Guide for Care and Use of Laboratory Animals, with antalgic, antibiotic and a recovery period.

#### Microdrives and recording setup

Four tetrodes formed a bundle threaded through a piece of stainless-steel tubing. Each tetrode consisted of four twisted $25\ \mu$m nichrome wires. The connector, tubing and wires could be moved downwards by turning the drive screw assemblies cemented to the skull. Cable was connected to the rat’s stage head, which contained a field effect transistor amplifier for each wire. The signals from each tetrode wire were amplified 10,000 times, bandpass filtered between 0.3 and 6 kHz with Neuralynx amplifiers (Neuralynx, Bozeman, MT, USA), digitised (32 kHz) and stored by the DataWave Sciworks acquisition system (DataWave Technologies, Longmont, CO, USA). A red light-emitting diode (LED) attached to the head assembly was used to determine the position of the rats. The LED was filmed by a CCD camera mounted on the ceiling above the maze, and their position was tracked at 50 Hz by a digital point tracker.

#### Behavioral T-maze training

The T-maze consisted of four 10 cm wide, grey-painted wooden tracks (with walls of 2 cm height on each side), a 100 cm long central rod, a 100 cm long crossbar forming the two choice arms, and two additional tracks each connecting the distal end of one choice arm to the base of the central rod. The reward wells were located at the distal end of each choice arm. Food rewards (45 mg sugar pellets) were dispensed from two food pellet dispensers (MedAssociates) mounted above the wells and activated by remote manual switches. The maze was elevated 40 cm off the ground on a metal frame. The apparatus was illuminated by four symmetrical spotlights (40 W) mounted on the ceiling. A centered radio above the maze was used to mask uncontrolled disturbing sounds and the experimenter was located in an adjacent room. After a recovery period from surgery, the rats were familiarized with the maze in daily 20-min sessions for two days, during which they were allowed to freely explore the apparatus and collect randomly scattered sugar pellets. Training began on the third day with two 20-min sessions per day. Rats had to run along the central rod and alternately enter the left or right arm of choice in order to obtain a 45 mg sugar pellet. Each rat performed one session per day as long as dorsomedial striatal units were clearly identified. Rats performed at least 18 sessions and at most 51.

#### Pre-processing of the behavioral data

A pre-processing of the data, consisting mainly in clearing rapid head movements and reflection artifacts of the camera, was performed and localization of the rats into a refined grid enabled us to identify which one of the elementary paths was taken (see Fig. 1b). Note that some rare portions of the recordings were not classified as one of the elementary paths. These was mostly due to the animals jumping out of the maze or jumping in a non contiguous spot in the maze. In any case, these portions were purely removed from the analysis.

#### Pre-processing of the neuronal data

Spike sorting was performed manually using the graphical cluster-cutting software Offline Sorter (Plexon). Units selected for analysis had to be well-discriminated cluster with spiking activity clearly dissociated from background noise. Units that were lost before the session series was completed, or whose waveform changed too much between two sessions, were not used for further analysis. Units having inter-spike intervals $<2$ ms (refractory period) were removed due to poor isolation, as were cells with a peak firing rate $\le 1$ Hz. A total of 293 cell clusters for the 4 rats was accepted and we assimilate them to neurons, each neuron being recorded in only one session (we do not try to track the waveform from one session to another). The datasets used and/or analysed during the current study available from the corresponding author on reasonable request.

In each session, each neuron produces a certain number of spikes in a certain region of the space in a given condition (for instance spikes produced in the central stem when it is part of a loop or a good path). To decide whether a neuron is coding in a particular region, we compare $N_1$ and $N_2$, the number of spikes produced during the session in the region of interest under condition 1 (resp. 2) (see Fig. 4a for the different regions and conditions). More precisely we compute the p-value of the chi-square test that decides if $(N_1,N_2)$ is a multinomial distribution of parameters $p=(d_1/(d_1+d_2),d_2/(d_1+d_2)$ and $n=N_1+N_2$, with $d_i$ the time spent in the region of interest under the condition *i* during the session. Therefore, for a given region and given pair of conditions, neurons were removed from the analysis if either one of the $d_i$’s were null (this depends solely on the behavior of the rat during the session) or if $np<5$ (this tends to eliminate neurons with very small average firing rate inside the region of interest). This gives the set of possible neurons (see Fig. 4a) and each possible neuron is linked to a pvalue. A possible neuron is declared to be coding in a particular region between two conditions if its adjusted p-value is less than 0.05. Here *adjusted p-value* refers to Benjamini and Hochberg method applied on all the possible neurons in a particular region between two given conditions.

### Specification of the CoACA models used in the T-maze experiment

The sequence of states during the experiment is formally denoted $S_{p,n,t}$: *p* is the session, going from 1 to *P*, *n* is the episode, going from 1 to $n_p$ (the number of episodes in session *p*), and *t* is the performed action, going from 1 to $T_{p,n}$ (the number of actions chosen in episode *n* and session *p*). All possible states are shown in Figure 2a.

At each state $S_{p,n,t}$, an action $A_{p,n,t}$ is performed to go to the next state $S_{p,n,t+1}$. The set of possible actions at a given state is formalized in Fig. 3 and depends on the model. During session *p*, at episode *n*, the rat obtains a reward $R_{p,n}$. If no good path is taken, $R_{p,n}=0$, if one good path is taken, $R_{p,n}=1$, and if the two good paths are taken, $R_{p,n}=2$ as shown in Figure 2b. At each episode *n*, a rat thus achieves a sequence $S_{p,n,1},A_{p,n,1},S_{p,n,2},A_{p,n,2},S_{p,n,3},...,,R_{p,n},S_{p,n,T_{p,n}}$.

All CoACA algorithms implement the same features. At session $p=1,...,P$, episode $n=1,...,n_p$ and action $t=1,...,T_{p,n}$, being in state $S_{p,n,t}$, the action $A_{p,n,t}$ is picked with probability defined in Eq. (2), with the credit $K_{p,n}$ defined recursively by Eq. (3), where $\alpha \in [0,1]$ the learning parameter to be fitted, $a(A_{n,t})= \frac{d(t)}{d(n)}$ the activity of action $A_{n,t}$, *d*(*t*) the duration of action *t*, *d*(*n*) the duration of episode *n*, and $R_{p,n}$ the reward obtained during session *p* and episode *n*. At initialization $K_{1,1}(S,A)=0$ for all state-action pairs (*S*, *A*). Notice that all state action pairs (*S*, *A*) that are not chosen in an episode are not updated: $K_{p,n+1}(S,A)= K_{p,n}(S,A)$.

2 $$\begin{aligned}&\mathbb {P}(A_{p,n,t}=A)= \frac{\exp (K_{p,n}(S_{p,n,t},A))}{\sum _{A'\text {possible from state } S_{p,n,t}}\exp (K_{p,n}(S_{p,n,t},A'))} \end{aligned}$$

3 $$\begin{aligned}&K_{p,n+1}(S,A) = K_{p,n}(S,A)+ \alpha \times \sum _{t=1}^{T_{p,n}} a(A_{n,t}) \textbf{1}_{A_{n,t}=A} \textbf{1}_{S_{n,t}=S} R_{p,n} \end{aligned}$$

To represent the decrease of action probabilities at each new session (see Fig. 1d), at the end of session *p*, all the credits are multiplied by forgetfulness parameter $\gamma \in [0,1]$:

4 $$\begin{aligned} K_{p+1,1}(S,A) = \gamma \times K_{p,n_p}(S,A) \end{aligned}$$

### Validation and application of the method

To validate our method, we follow the 10 Rules defined in^24^. The design of experiment (Rule 1) has been done in subsection “Experimental design” and the design of models (Rule 2) in subsection “Specification of the CoACA models used in the T-maze experiment”.

#### Simulations of the models

We use the Rcpp package (https://cran.r-project.org/web/packages/Rmpi/index.html, lastly accessed on 07-13-2022) for efficiency reasons. The simulations were run fully in parallel using Rmpi package(https://cran.r-project.org/web/packages/Rmpi/index.html, lastly accessed on 07-13-2022). The NEF computing platform ( https://wiki.inria.fr/ClustersSophia/Usage_policy, lastly accessed on 07-13-2022) has been used for running the parallel simulations. The platform has 320 Xeon SP Silver 2.2GHz cores, 128 Xeon E5 v2 2.6GHz cores and 320 Xeon E5 v2 2.8GHz cores.

Both validation and estimation are computationally intensive. For the likelihood estimation of each model, 50 Xeon cores were used for an execution time of approximately 1 hour for each rat. For the hold-out part, 200 Xeon cores were used for an execution time of approximately 6 hours for each rat.

Maximum Likelihood Estimators (MLE) are computed as explained in^23,24^ thanks to the function DEoptim of R applied on minus the log-likelihood. To avoid local minima, the optimisation algorithm was randomly initialized 200 times.

For Rule 3 (Simulation of the models), we simulated the different models (i) with parameters chosen uniformly on $\hat{\theta }(1 \pm 0.05)$, where $\hat{\theta }$ are the MLE parameters estimated on the real data and (ii) with a number of actions per session equal to the ones observed on real data of each given rat. The simulation of a given model does not always reach the Learning Criterion (LC), that is 80% of good paths in one session (criterion that is matched by all the rats in the experiments. Such simulations were discarded and we evaluated the performance of the MLE only on the simulations reaching LC. However for some models, it was difficult even after 10000 runs to get 20 simulations reaching LC (see Supplementary Fig. S1). Note that Hybrid 3, always meets the learning criterion in a consequent number of simulations.

To check the efficiency of the MLE (Rule 4–5), one computes, for each simulation that met the learning criterion, the estimator as a function of the number of paths that were taken into account in the MLE procedure. Note that we chose paths and not actions as a unit since the number of actions depend on the model. Supplementary Fig. S1 shows that the MLE converges for the models that are meeting the learning criterion, even if it is much slower for the forgetfulness parameter $\gamma$ since one needs to see several sessions to estimate it correctly. Note that after about 800 paths the MLE’s are quite close to the model parameters.

The choice of 800 paths for the cut between early learning phase and late learning phase in the hold-out method is further validated (Rule 6) by exhibiting the confusion matrix for this precise choice (see Supplementary Fig. S2 where the simulation is done with the MLE estimators on real data on the whole data set). One can see that except for rat 2 with the model Path which has only 67 % of correct recognition, all other cases achieve 83% when the model reaches LC in sufficiently many simulations.

#### Real data analysis

On real data (Rule 7), the stability of the MLE as a function of the number of Paths (Supplementary Fig. S3) seems good in most cases after 800 paths, except for Rat 2 for which 800 might seem slightly too small. However to simplify procedures, we decided to take the same cut at 800 paths for all rats.

To validate the winning model (Rule 8), we represented the vector of the probabilities of each path during an episode as a function of time and reduced its dimensionality by Principal Component Analysis. This representation (see Supplementary Fig. S4) shows that even if the empirical choice probability of the different paths is noisier than the one given by the models, Hybrid 3 seems most of the time to represent well what is happening at least in the late learning phase.

For the record, Rule 9 consists in analyzing the winning model with respect (for instance) to physiological data and this is what we did in Fig. 4 and Rule 10 consists in reporting the analysis.

### Supplementary Information

Supplementary Information.
