# Author Correction: Strategy inference during learning via cognitive activity-based credit assignment models

**DOI:** 10.1038/s41598-023-40524-8

**Published:** 2023-08-15

**Authors:** Ashwin James, Patricia Reynaud-Bouret, Giulia Mezzadri, Francesca Sargolini, Ingrid Bethus, Alexandre Muzy

**Affiliations:** 1 Université Côte d’Azur, CNRS, I3S, Nice, France; 2 Université Côte d’Azur, CNRS, LJAD, Nice, France; 3 Columbia University, Cognition and Decision Lab, New York, USA; 4 Aix Marseille Université, CNRS, Laboratoire de Neurosciences Cognitives, Marseille, France; 5 Université Côte d’Azur, CNRS, IPMC, Nice, France

Correction to: *Scientific Reports* 10.1038/s41598-023-33604-2, published online 09 June 2023

In the original version of this Article Alexandre Muzy was incorrectly affiliated with ‘Université Côte d’Azur, CNRS, IPMC, Nice, France’. The correct affiliation is listed below.

Université Côte d’Azur, CNRS, I3S, Nice, France

Additionally, the Acknowledgements section contained errors.

“This work was supported by the French government, through the ${\text{UCA}}^{\text{Jedi}}$ and 3IA Côte d’Azur Investissements d’Avenir managed by the National Research Agency (ANR-15- IDEX-01 and ANR-19-P3IA-0002), by the interdisciplinary Institute for Modeling in Neuroscience and Cognition (NeuroMod) of the Université Côte d’Azur and directly by the National Research Agency (ANR-19-CE40-0024) with the ChaMaNe project. It is part of the Computabrain project.”

now reads:

“This work was supported by the French government, through the ${\text{UCA}}^{\text{Jedi}}$ and 3IA Côte d’Azur Investissements d’Avenir managed by the National Research Agency (ANR-15- IDEX-01 and ANR-19-P3IA-0002), by the interdisciplinary Institute for Modeling in Neuroscience and Cognition (NeuroMod) of the Université Côte d’Azur, by the National Research Agency (ANR-19-CE40-0024) with the ChaMaNe project and by the Projet de Recherche Inter-instituts Multi-Equipes (80|PRIME) CNRS. It is part of the Computabrain project and the eXplAIn CNRS research team.”

The original Article has been corrected.

