# Supplementary material for: Strategy inference during learning via cognitive activity-based credit assignment models
Source: Sci Rep. 2023 Jun 9;13:9408. doi: 10.1038/s41598-023-33604-2 (PMC10256696; doi:10.1038/s41598-023-33604-2)
Supplement: Supplementary file 1 — Supplementary Information. [file 41598_2023_33604_MOESM1_ESM.pdf]

Supplementary Information

Ashwin James, Patricia Reynaud-Bouret, Giulia Mezzadri, Francesca Sargolini, Ingrid Bethus, Alexandre Muzy

ABSTRACT

This PDF file includes:

- Fig. S1: Stability of Maximum Likelihood Estimation
- Fig. S2: Confusion Matrix
- Fig. S3: MLE on Real Data
- Table 1: Simulation Parameters
- Table 2: Rat sessions
- Fig. S4: Principal Component Analysis

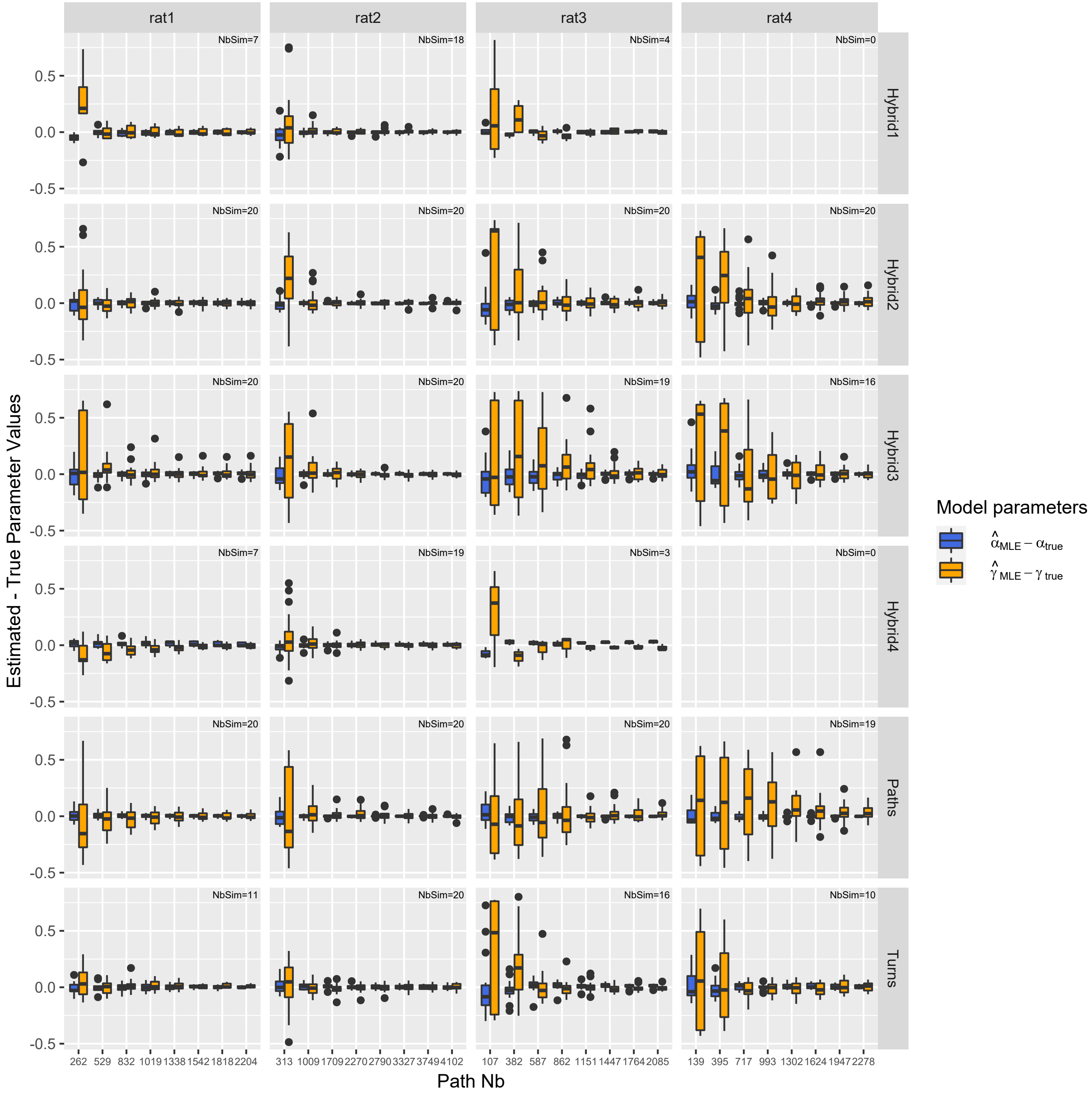

Figure S1. Boxplots of the difference between the Maximum Likelihood Estimators and the model parameters as a function of the number of paths used for the MLE computation. The model parameters are selected uniformly in the the range $\alpha_{true}(1 \pm 0.05), \gamma_{true}(1 \pm 0.05)$ and then used for simulation. NbSim indicates the number of simulations used to compute the boxplots. NbSim < 20 indicates that for the model under consideration the number of simulations meeting the learning criteria (80 % of good paths in at least one session) was less than 20 after generating 10000 simulations.

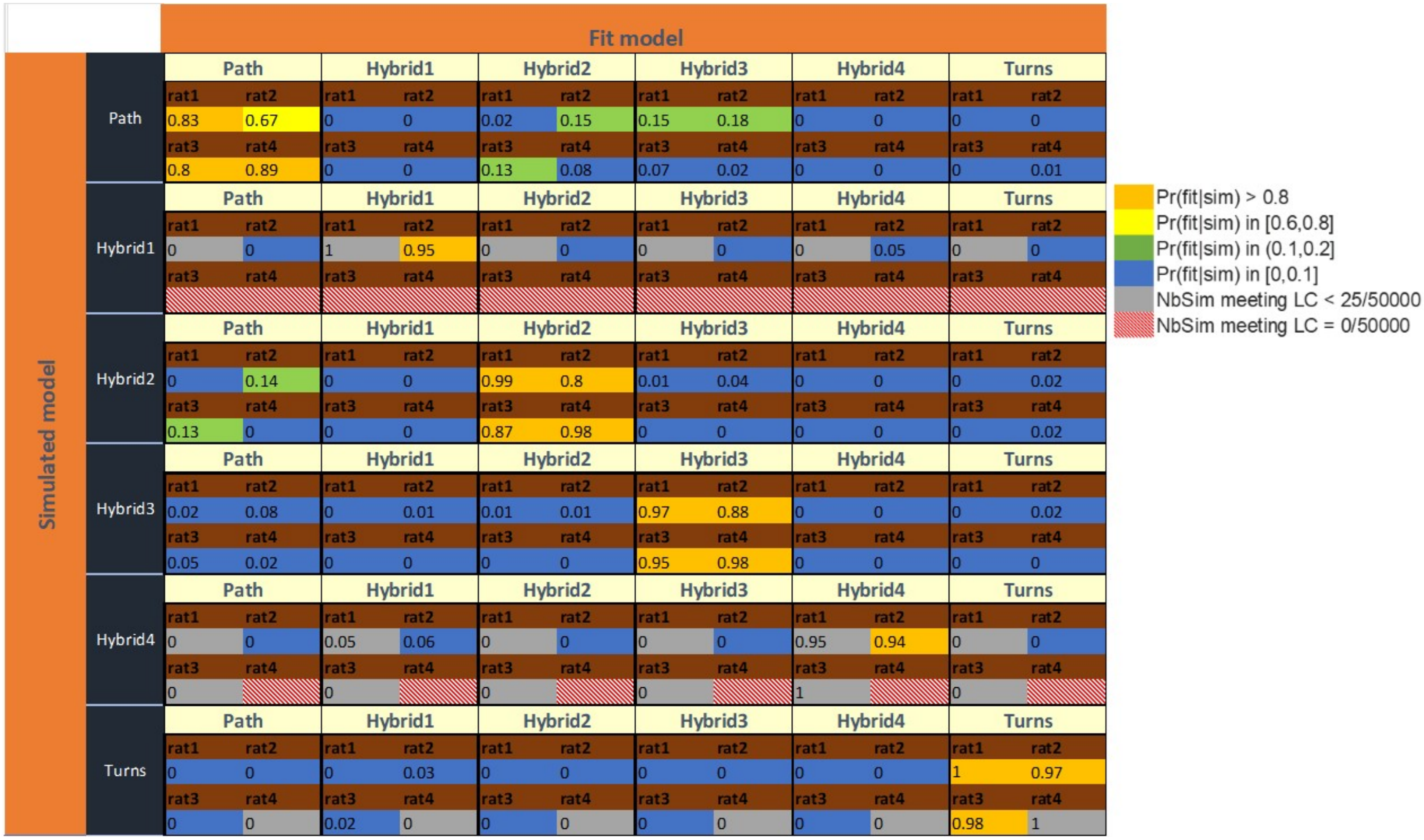

Figure S2. Confusion matrix showing $Pr(fit\ model = B|simulated\ model = A)$. The fit model is estimated via the hold-out method with a cut between early and late learning phase at 800 paths. Simulations are generated with the model parameters estimated by MLE on the experimental data of rats in Figure ??. Each simulation has the same number of sessions and paths per session as in the real experiment. Only the simulations reaching the Learning Criteria (LC) (80% of good paths in at least one session) are used to construct the confusion matrix. The probability matrix was computed using 100 simulations in each case except when the Number of Simulations meeting LC out of 50000 runs was less than 25, in which case the average is done on the number of simulations reaching LC. When no simulations met LC, the quantity is not computed.

Table 1. Simulation Parameters. The α and γ parameter values obtained by maximum likelihood estimation on real data from each rat used to generate simulated data using CoACA.

Rat	Paths	Hybrid1	Hybrid2	Hybrid3	Hybrid4	Turns
rat1	α=0.13,γ=0.37	α=0.21,γ=0.28	α=0.21,γ=0.37	α=0.23,γ=0.38	α=0.2,γ=0.26	α=0.34,γ=0.3
rat2	α=0.09,γ=0.41	α=0.24,γ=0.29	α=0.14,γ=0.46	α=0.16,γ=0.44	α=0.23,γ=0.3	α=0.27,γ=0.42
rat3	α=0.09,γ=0.32	α=0.2,γ=0.2	α=0.16,γ=0.31	α=0.18,γ=0.31	α=0.19,γ=0.18	α=0.29,γ=0.25
rat4	α=0.07,γ=0.42	α=0.11,γ=0.34	α=0.12,γ=0.41	α=0.13,γ=0.4	α=0.11,γ=0.33	α=0.18,γ=0.36

Table 2. Rat sessions: The number of paths in each experimental session for all rats. The simulated data for each rat was generated with the same number of paths in each session as the experimental data.

Rat	Session Lengths
rat1	110, 52, 100, 95, 120, 52, 129, 94, 80, 63, 42, 82, 128, 99, 92, 133, 22, 49, 112, 74, 90, 94, 158, 134 (24 sessions)
rat2	48, 55, 99, 111, 121, 138, 136, 148, 153, 150, 164, 121, 134, 131, 119, 140, 128, 174, 122, 118, 127, 153, 107, 159, 140, 131, 68, 94, 123, 137, 97, 27, 108, 121 (34 sessions)
rat3	49, 58, 66, 91, 118, 96, 109, 146, 129, 140, 149, 152, 144, 161, 156, 149, 172 (17 sessions)
rat4	55, 84, 74, 97, 85, 96, 97, 129, 142, 134, 157, 152, 157, 165, 166, 157, 158, 173 (18 sessions)

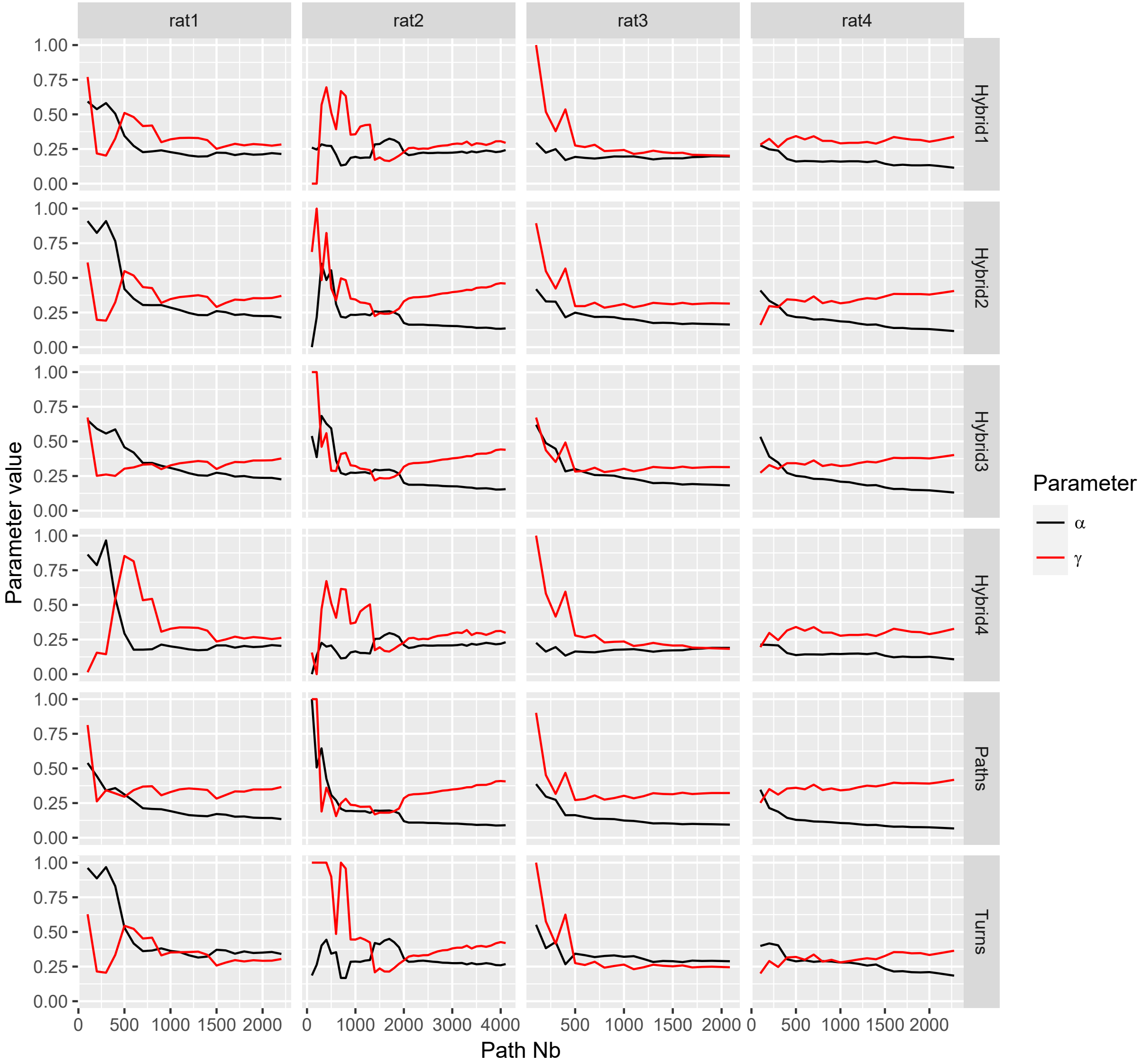

Figure S3. MLE on real data. For each rat and each model m, the MLE estimators $(\hat{\alpha}_m^{MLE}, \hat{\gamma}_m^{MLE})$ are plotted as a function of the number of paths (estimation was done every 100 paths until the end of the data).

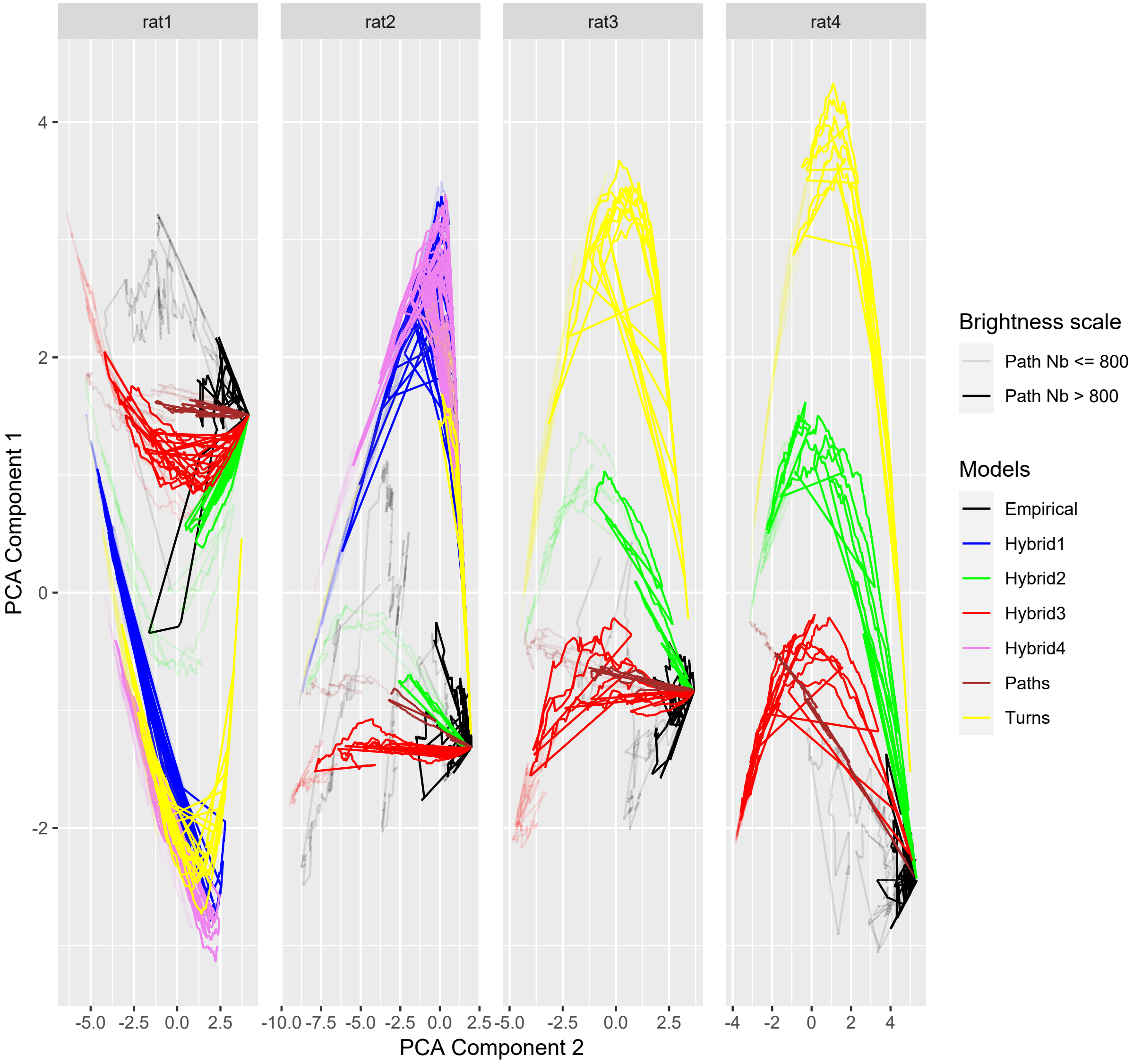

Figure S4. Representation of the probabilities of the different paths in the first plane of Principal Component Analysis (PCA). The probabilities of the simulated CoACA models with the model parameters given in Figure **??** are compared with the empirical probabilities computed on the real data. The probabilities of the simulated models of all the 12 possible paths in the maze were concatenated into a probability matrix with 12 rows. The empirical path probabilities for each of the 12 paths (computed as a centered moving average over 40 paths inside the same session on the experimental data) were also added to the above matrix. This probability matrix was then used to generate to compute the PCA for each rat.
